# The Prevalence of Previously Undiagnosed Leprosy in the General Population of Northwest Bangladesh

**DOI:** 10.1371/journal.pntd.0000198

**Published:** 2008-02-27

**Authors:** Fake J. Moet, Ron P. Schuring, David Pahan, Linda Oskam, Jan Hendrik Richardus

**Affiliations:** 1 Department of Public Health, Erasmus MC, University Medical Center Rotterdam, Rotterdam, The Netherlands; 2 KIT (Koninklijk Instituut voor de Tropen/Royal Tropical Institute), KIT Biomedical Research, Amsterdam, The Netherlands; 3 Rural Health Program, The Leprosy Mission Bangladesh (formerly DBLM), Nilphamari, Bangladesh; London School of Hygiene and Tropical Medicine, United Kingdom

## Abstract

**Background:**

The prevalence of previously undiagnosed leprosy (PPUL) in the general population was determined to estimate the background level of leprosy in the population and to compare this with registered prevalence and the known PPUL in different levels of contacts of leprosy patients.

**Methodology and Principal Findings:**

Multistage cluster sampling including 20 clusters of 1,000 persons each in two districts with over 4 million population. Physical examination was performed on all individuals. The number of newly found leprosy cases among 17,862 people above 5 years of age from the cluster sample was 27 (19 SLPB, 8 PB2-5), giving a PPUL rate of 15.1 per 10,000.

**Conclusions and Significance:**

PPUL in the general population is six times higher than the registered prevalence, but three times lower than that in the most distant subgroup of contacts (neighbour of neighbour and social contacts) of leprosy patients in the same area. Full village or neighbourhood surveys may be preferable to contact surveys where leprosy is highly endemic.

## Author Summary

In order to estimate the level of leprosy in an area with many leprosy patients, we determined the prevalence of previously undiagnosed leprosy in the general population and compared this with the registered (or known) number of leprosy patients. We also compared it with the known prevalence of leprosy in contacts of leprosy patients. We examined 20 randomly selected geographical clusters of 1,000 persons each in two districts of Bangladesh, with over 4 million population. Physical examination was performed on all individuals. The number of newly found leprosy cases among 17,862 people above 5 years of age from the clusters was 27, giving a rate of previously undiagnosed leprosy of 15.1 per 10,000. This rate is six times higher than the registered prevalence, but three times lower than the rate in the most distant subgroup of contacts (neighbour of neighbour and social contacts) of leprosy patients in the same area. We conclude that in areas where leprosy is common, it may be preferable to do full village or neighbourhood surveys when a new leprosy patient is found, rather than to limit contact surveys to close contacts only, such as household members.

## Introduction

For over 60 years it is known that contacts of leprosy patients have a higher risk of developing leprosy than people in the general population.[1] Besides the type of leprosy of the index patient, i.e. multibacillary (MB) leprosy, the physical distance is also an important factor determining this risk.[2] It is likely that, as the distance increases, the relative risk for having leprosy as compared to the general population gradually comes down to one. Contact examination is an important intervention strategy to find early leprosy cases among close contacts of recently diagnosed leprosy patients, but it is unclear to what level of contact this is effective in terms of preventing new cases of leprosy and transmission of *M. leprae* in the population. Therefore it is important to know the background prevalence of leprosy in the population.

As part of a larger study into transmission of *M. leprae* and the possibility to target contacts with preventive interventions such as chemoprophylaxis[3], we estimated the background prevalence of leprosy in an endemic community through a random sample of the general population. The aim of the study was to establish the prevalence of previously undiagnosed leprosy (PPUL) in the general population and compare this with the registered prevalence of leprosy, and with the prevalence of PPUL among different levels of contacts of leprosy patients in the same population.

## Methods

The study population consisted of the inhabitants of the Rangpur and Nilphamari districts in northwest Bangladesh. The total population is over four million people (estimated population in 2000, based on the 1991 census). The registered new case detection rate of leprosy in this part of the country was 3.21 per 10,000 in 2002 (DBLM Annual Report 2002). This figure is based primarily on passive case detection (self-referral or referral by local health workers: 74%), and also active detection methods such as household contact, school and village surveys (26%). Out of the total population a random sample was taken to estimate the prevalence of previously undiagnosed leprosy (PPUL), and the leprosy control staff of the Rural Health Program (formerly DBLM) of The Leprosy Mission Bangladesh performed active door-to-door screening. As leprosy is known to occur in clusters, one large sample from a single area may not have given a reliable approximation of the leprosy situation in the two districts, so more samples had to be taken from different areas. Therefore a multistage cluster sampling procedure as described in literature was followed.[4]

### Sampling procedure

A total of 20 clusters of 1000 people each were randomly sampled from the 13 sub-districts (thana's). One to three clusters were allocated to each sub-district proportionally to the size of its population. A list of unions (in rural areas) and wards (in urban areas) per sub-district was drawn up. A union or ward has an average population of around 23,500. In case the population of a large union was more than three times the size of that of the smallest union, the largest union was split. Then one to three unions (the number of clusters allocated to that sub-district) were selected from the list by means of computerised randomisation. Per selected union a list of all sub-unions (mostly equivalent to villages) was prepared in such a way that the population of the largest village was maximally three times the population of the smallest. These sub-unions have an average population of 5300. Grouping of small villages was sometimes needed, as the accepted minimum size was a population of 1600 (estimation based on census 1991). The computer then randomly selected one sub-union per union. Three out of the twenty clusters were thus allocated to urban areas, which is a proper reflection of the population figures.

### Survey

The surveys of all clusters were performed between November 2002 and February 2003. The population of the village/area was informed in advance about the purpose and time the team would perform the survey. During the survey the people were asked about symptoms of leprosy and a body check was performed. Genital areas, and for females also the buttocks and the breasts, were not examined. The survey included all people present, whereby female health workers examined the adult females. It started at the northern border of the selected area and stopped when about 1000 people were examined. The criteria used for diagnosis and classification were those of the local leprosy control programme, which follows the WHO guidelines [5], but those patients with a single lesion with a satellite were recorded as single lesion paucibacillary (SLPB) and not as paucibacillary with 2–5 lesions (PB2-5).[6] All persons suspected of having leprosy were referred to an experienced medical doctor for confirmation. If the disease was confirmed, people were offered regular treatment. All data were entered on registration cards, whereby partly filled cards were used for the next household.

### Analysis

Data were analysed by means of descriptive statistics and logistic regression with the Statistical Package for the Social Sciences (SPSS for Windows, release 11.0.1, SPSS Inc., Chicago, Illinois).

### Ethical clearance

We obtained ethical clearance from the Ethical Review Committee of the Bangladesh Medical Research Council in Dhaka (ref. no. BMRC/ERC/2001-2004/799). All subjects were informed verbally in their own language (Bangla) about the study and invited to participate. Written consent was requested from each adult. For children consent from a parent or guardian was given.

## Results

The total number of people enumerated on the registration cards was 20,299 of whom 100 were excluded because there were missing data in the records. Of 52 people it was known that they were released from leprosy treatment (RFT) before the survey. As cured leprosy patients presumably can become infected again, these known RFT cases were not excluded. There were 2337 children (1208 male and 1129 female) below the age of five years. As we used the figures in comparison to the figures from the COLEP chemoprophylaxis trial from which under-fives were excluded[3], the children below the age of five were also excluded from the analysis in this study. This left 17,862 persons for this analysis. Table 1 shows the sex and age distribution by cluster. Among these people, 27 previously undiagnosed cases of leprosy were found. The PPUL is thus 15.1 per 10,000 (95% CI = 9.4–20.8). All newly found cases had PB leprosy (19 SLPB, 8 PB2-5). None of the children younger than 5 years of age had leprosy, so when they are included, the PPUL comes down to 13.4 per 10,000.

**Table 1:** Sex, age, newly found leprosy patients by cluster.

Cluster	N	M/F ratio^1^	Age				No. of newly found cases	Newly found cases per 10,000	Registered prevalence^2^
			Mean	25^th^ percentile	50^th^ percentile	75^th^ percentile			
1	938	0.70	25.9	11	23	36	0	0	4.91
2	895	0.70	24.2	11	19	35	6	67.0	4.26
3	871	0.99	25.5	11	21	36	0	0	2.51
4	866	0.59	25.9	11	23	37	0	0	2.03
5	897	0.73	29.1	13	25	43	0	0	2.03
6 (urban)	904	0.53	23.4	11	20	33	2	22.1	3.42
7	852	0.64	25.3	11	23	36	1	11.7	3.42
8	892	0.73	26.1	12	21	38	5	56.1	4.21
9	934	0.85	27.0	13	23	36	0	0	1.71
10	911	0.58	27.4	12	24	41	3	32.9	3.98
11	862	0.55	25.1	11	23	35	0	0	1.45
12 (urban)	862	0.72	26.5	11	23	38	4	46.4	1.61
13 (urban)	913	0.68	26.4	13	23	38	1	11.0	1.61
14	903	0.92	28.3	13	27	41	0	0	1.61
15	848	0.58	30.0	14	26	41	0	0	0.91
16	950	0.81	28.2	13	26	41	1	10.5	0.91
17	934	0.63	28.4	13	26	41	1	10.7	0.91
18	872	0.59	28.8	15	26	40	3	34.4	0.99
19	865	0.68	27.4	13	25	38	0	0	0.99
20	893	0.69	26.2	11	23	38	0	0	1.30
Total	17,862	0.69	26.8	12	23	38	27	15.1	2.31
								(CI 95% 9–21)	

1 M/F ratio = male/female ratio.

2 Registered prevalence (at sub-district level) per 10,000 population per September 30, 2002, before the survey.

Table 2 shows the PPUL per age group and by sex. There is no difference in risk between the sexes, but there is a trend that people of higher age are more at risk. When the subjects are divided into two age groups (under 30 years of age and 30 years and above), age is a statistically significant risk factor. The OR for those 30 years of age or older is 2.55 (95% CI = 1.17–5.57, p = 0.019).

**Table 2:** Number of people examined and prevalence of previously undiagnosed leprosy (PPUL) per 10,000 by age and sex.

Age (in years)	Male			Female			Total PPUL
	N	Leprosy	PPUL	N	Leprosy	PPUL	
5–9	1542	1	6.5	1597	0	0	3.2
10–14	1277	2	15.7	1378	2	14.5	15.1
15–19	746	1	13.4	1115	1	9.0	10.7
20–29	963	0	0	2091	3	14.4	9.8
30–39	979	4	50.6	1964	2	10.2	20.4
40–49	797	2	25.2	1279	3	23.5	24.1
≥50	973	1	10.3	1159	5	43.2	28.1
Not recorded	1	0	0	1	0	0	0
Total	7278	11	15.1	10584	16	15.1	15.1

## Discussion

The PPUL in northwest Bangladesh in the population of 5 years and older, as found by means of a random cluster survey, is 15.1 per 10,000. This study, which included about 0.5% of the total population of the area, was based on established multistage cluster sampling techniques. We believe that the results give a reliable picture of the leprosy situation in northwest Bangladesh, in an area where an extensive leprosy control programme has been implemented for more than 10 years.

Potential sources for selection and information bias were considered, especially as only those present during the survey were included. Selection bias on cluster level is not likely, but on individual level selection bias is possible as the survey is announced in advance and those afraid of the diagnosis may go into hiding. Males are less likely to be at home during the day and indeed only 42% of those examined are males. In our data, however, the PPUL among males and females is the same. It is possible that, due to stigma, those with leprosy have a higher chance of being unemployed or rejected at school, so they could be over-represented at the survey, but as all patients found were in the early stage of the disease, this does not seem to be a likely reason for the high number of cases found in our study. We conclude that the possible sources of bias probably have had no effect.

In the past, over-diagnosis has not been a problem in this particular field programme, as was confirmed by an independent evaluator in 2001[7], but to avoid possible over-diagnosis in this study, all suspected cases were seen by senior leprosy control officers with more than 5 years experience in the diagnosis of leprosy at referral centre level, and confirmed by a medical doctor.

We found that the PPUL (including children under five) found by active screening was nearly 6 times higher than the registered prevalence (13.4 vs. 2.31/10,000). Registered prevalence is largely based on passive case detection. A large difference between the official new case detection (NCD) or prevalence, based on passive case detection, and the NCD or prevalence found by door-to-door surveys has been described before. For example, Schreuder et al. found by a rapid village survey in Java, Indonesia, two and a half times the number of known cases[8], and Bakker et al. found during a survey on a few small Indonesian islands 96 cases of leprosy of whom only 11 were previously known.[9] Different sample surveys in India have also revealed sample prevalences 4–5 times the recorded prevalence.[10] Self-healing of leprosy contributes to the difference between active and passive case-finding. In South India Ekambaram et al. found that the percentage of self-healing among non-lepromatous patients was around 74%.[11] In Africa Browne found that 34% of non-treated patients healed spontaneously.[12]

Table 3 shows the PPUL in the general population sample as described in this paper, together with the PPUL in the subgroups of contacts of leprosy patients as found during the intake of the COLEP trial.[13] These subgroups were defined by their physical distance to the index patient. The age distribution in the general population examined is similar to the distribution in the contact group, so this is not a major cause for bias. In the contact group of the COLEP study as a whole, the PPUL rate was 73/10,000, compared to 15.1/10,000 in the population sample.[3],[13] With regard to the different categories in the contact group, we conclude that even in the most distant category (the neighbours of the neighbours and social contacts) the PPUL rate (49/10,000) does not come down to the same level as that of the general population. It may therefore be preferable under such high-endemic circumstances to conduct full village or neighbourhood surveys instead of (close) contact surveys.

**Table 3:** Prevalence of previously undiagnosed leprosy (PPUL) per 10,000 in the subgroups of the contact population of the COLEP study [13] and in the general population sample.

Source of information	Leprosy contacts and general population	PPUL	95% CI^1^
COLEP study [13]	Sharing kitchen and roof (“household”)	156	106–220
	Sharing kitchen only	75	39–131
	Sharing roof only & next-door neighbour, not sharing roof or kitchen	87	65–115
	Neighbour of neighbour & social contact	49	38–63
This study	General population sample	15	9–21

1 95% CI = 95% confidence interval for PPUL.

There is a marked variance in PPUL among the different clusters. A gradient along geographical lines was not found. The clusters with a low number of newly found cases are scattered over both districts, as are the clusters with the highest numbers. In the three urban clusters however, relative high numbers of cases were found. This is in contrast to the findings of Kumar et al. in Agra, India, where the prevalence of leprosy in the urban areas was about one third lower than in the rural areas.[14] Sterne et al. observed a lower incidence of leprosy in the semi-urban district capital of the Karonga District in Malawi[15], while Lapa et al. report that in the State of Pernambuco, Brazil, leprosy is mainly an urban disease.[16]

In conclusion, our data show that the PPUL in the general population is six times higher than the registered prevalence, but three times lower than that in the most distant subgroup of contacts of leprosy patients in the same area. It has to be kept in mind however, that most new cases in populations where leprosy is relatively highly endemic come from the non-close contact group. Hence full village or neighbourhood surveys might be preferable to contact surveys under such circumstances.[17] There are indications that in lower endemic areas the incidence of leprosy among contacts declines faster as the physical distance to the patient increases.[18] If that is indeed the case, screening of contacts further removed from the patient might not be as useful in lower endemic areas.
